# A case of *Enterococcus cecorum* in a renal transplant recipient and literature review

**DOI:** 10.1007/s10096-025-05190-w

**Published:** 2025-07-29

**Authors:** Valli Mani, Ilan Berlinrut, Frances Wallach, Esther Benamu Sultan, Mersema Abate

**Affiliations:** 1 Department of Medicine, Donald and Barbara Zucker School of Medicine at Hofstra/Northwell, Hempstead, USA; 2 Dayton and Karen Brown Division of Infectious Diseases, Donald and Barbara Zucker School of Medicine at Hofstra/Northwell, Hempstead, USA; 3 Division of Nephrology, Donald and Barbara Zucker School of Medicine at Hofstra/Northwell, Hempstead, USA

**Keywords:** Enterococcus cecorum, Renal transplant, Immunocompromised, Poultry

## Abstract

*Enterococcus cecorum* is a bacterium typically found in the gastrointestinal tract of chickens, with rare cases reported in humans. We present a case of *Enterococcus cecorum* bacteremia in a renal transplant recipient with possible source of pet parrots. We also review the literature on *E. cecorum* infection in humans.

## Introduction

*Enterococcus cecorum* is a gram-positive coccus isolated from the gastrointestinal tracts of farm animals, particularly poultry [1]. Unlike *E. faecalis* or *E. faecium*, *E. cecorum* is not a commensal species found in the human intestinal tract [1]. There are less than 10 cases reported of *E. cecorum* infection in humans and only one prior case in the United States [2]. The patients reported developed severe complications including septicemia [3], endocarditis [4], empyema [5], and peritonitis [6]. Most had underlying immune impairing medical conditions that predisposed them to infection [7]. We present a patient who was found to have *E. cecorum* bacteremia 14 years after renal transplantation.

## Case presentation

A 74-year-old female with a past medical history of autosomal dominant polycystic kidney disease status post live donor kidney transplant in 2009 on multiple immunosuppressives (mycophenolate, tacrolimus, and prednisone), hypertension, gastroesophageal reflux disease, and frequent urinary tract infections was admitted to the hospital with three weeks of subjective fevers, myalgias, diarrhea, and cough. She reported these symptoms and a positive COVID-19 test since returning from a cruise two weeks prior to presentation. She was an avid bird lover and the caretaker of multiple pet parrots.

Upon presentation, she was afebrile and hemodynamically stable, with a white blood cell count of 4,000 × 10 [7] L. Notable labs included elevated creatinine of 2.37 mg/dL (baseline of 0.9 mg/dL), positive COVID-19 PCR test, and urine culture with 50 k-99 k *Escherichia coli*. Gastrointestinal panel PCR and stool culture were negative. Remdesivir was started for COVID-19 infection, without concurrent antibiotic therapy.

Two days after admission, two sets of blood cultures grew *Enterococcus cecorum.* She was prescribed Ampicillin 2 g every 6 h (S < 2) with persistently positive blood culture in one of four bottles after 3 days of therapy. The infectious disease team decided to add Ceftriaxone 2 g every 12 h (susceptibility pattern not reported), citing evidence for use of this combination in severe Enterococcal infections, including endocarditis. The next set of cultures obtained two days later showed no growth. Susceptibility testing of the isolate revealed sensitivity to most antibiotics tested, including Ampicillin, Vancomycin, Linezolid, Tetracycline and Ciprofloxacin. CT imaging of the abdomen and pelvis did not show a source of bacteremia; however, contrast was not administered. Transesophageal echocardiogram did not show valvular vegetations. After the patient was stabilized, she was discharged home on Linezolid to complete a two-week course of antibiotics from negative blood cultures.

## Discussion

Though there are only a few case reports detailing infection with *Enterococcus cecorum* in humans, pathogenic strains of this bacterium have been causing significant morbidity and mortality in chickens over the past 20 years. Transmission of this organism is not entirely understood; however, in infected poultry, it is through direct contact or exposure to contaminated feces [8].

Our patient initially presented to the hospital with a vague constellation of symptoms attributed to COVID-19. However, infectious workup revealed *Enterococcus cecorum* bacteremia. While avian outbreaks have been linked to chickens, susceptibility to this infection has been observed in ducks and pigeons, broadening the potential reservoirs beyond traditional poultry [9]. This woman possibly contracted *E. cecorum* through exposure to feces from her pet parrots; however, stool molecular testing on birds was not performed to say with certainty.

Among the cases of *Enterococcus cecorum* infection reported in the literature, decompensated cirrhosis was the most common underlying condition [5]. The gastrointestinal tract served as the likely port of entry, with intestinal mucosal edema and local immunosuppression from portal hypertension contributing to systemic infections, such as bacteremia, peritonitis, and empyema. Other immunocompromising conditions in patients included asplenia, chronic myelogenous lymphoma, and Crohn’s disease [7]. One case reported a patient who, one-year post-renal transplantation, was found to be colonized with *E. cecorum* on routine in-office urine culture [7]. Antibiotics were not administered, as this was not deemed a clinically relevant infection.

Though our patient received a kidney transplant 14 years ago, she was taking multiple immunosuppressive medications. In particular, mycophenolate can damage the intestinal epithelial barrier through disruption of tight junctions and apoptosis [10]. Increased gut permeability likely facilitated translocation of this pathogen from the gastrointestinal tract to the bloodstream [11]. Fortunately, this bacterium was sensitive to multiple antibiotics and the infection was controlled with Ampicillin and Ceftriaxone. However, Enterococcus spp. often exchange conjugative plasmids and transposons across various species and genera, making *E. cecorum* an important reservoir for antimicrobial resistance in the future. Therefore, in immunocompromised individuals with bird exposure, clinicians should have increased suspicion for *Enterococcus cecorum* infection in order to ensure timely diagnosis and management.

## Data Availability

No datasets were generated or analysed during the current study.
